# Supplementary material for: Causal Relationship between Aging and Anorexia Nervosa: A White-Matter-Microstructure-Mediated Mendelian Randomization Analysis
Source: Biomedicines. 2024 Aug 16;12(8):1874. doi: 10.3390/biomedicines12081874 (PMC11351342; doi:10.3390/biomedicines12081874)
Supplement: Supplementary file 1 [file biomedicines-12-01874-s001.zip › Supplementary Figure/Figure S8/Leaveoneout plot of MR analysis for TL to ubm-b-1455.pdf]

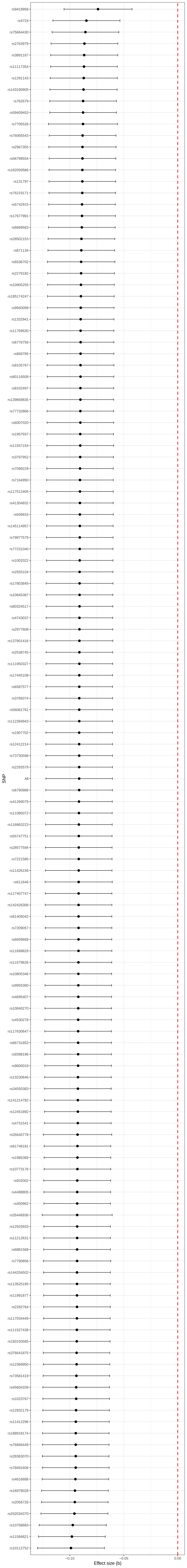
rs9419958
rs4724
rs75664430
rs2763979
rs3891167
rs11117354
rs1291143
rs143190905
rs762679
rs59409453
rs7705526
rs76065543
rs2967355
rs56799554
rs182059586
rs131797
rs76219171
rs5742915
rs17677991
rs6669563
rs28502153
rs871134
rs6536702
rs2276182
rs10905255
rs185174247
rs9940099
rs1332941
rs11769630
rs6776756
rs869785
rs8105767
rs80116508
rs8102497
rs139669835
rs77732866
rs6007020
rs1957937
rs11557154
rs3767952
rs7099229
rs7164950
rs117512405
rs41304832
rs939916
rs145114957
rs79977579
rs77231040
rs1003322
rs2555104
rs17803849
rs10845387
rs80324517
rs4743037
rs2977608
rs137901416
rs2538745
rs111950327
rs17445108
rs6587577
rs3785074
rs56061761
rs112394453
rs1907702
rs12412214
rs73730598
rs2293579
A8
rs6790988
rs41269079
rs11085072
rs116863223
rs55747751
rs28577594
rs7221585
rs11426156
rs611646
rs117407747
rs142426306
rs61405042
rs7209057
rs6659669
rs11699829
rs11579626
rs10805346
rs9955360
rs4695407
rs10840270
rs4530278
rs117630647
rs66731853
rs9398196
rs9600019
rs13230646
rs34550383
rs141214782
rs12451892
rs4731541
rs35640778
rs61748181
rs1985369
rs10773176
rs932002
rs4498805
rs450962
rs35446936
rs12925933
rs11212631
rs6081568
rs7790856
rs144204502
rs113525195
rs11991877
rs2282764
rs117034449
rs111527438
rs150150565
rs376641875
rs12369950
rs73581419
rs45604339
rs1023767
rs12932179
rs11412296
rs188918174
rs76066448
rs28363070
rs78491606
rs4616688
rs16978028
rs2056726
rs202034370
rs10768683
rs11584821
rs10112752
SNP
-0.10
-0.05
0.00
Effect size (b)
